# Task Similarity-Aware Cooperative Computation Offloading and Resource Allocation for Reusable Tasks in Dense MEC Systems

**DOI:** 10.3390/s25103172

**Published:** 2025-05-17

**Authors:** Hanchao Mu, Shie Wu, Pengfei He, Jiahui Chen, Wenqing Wu

**Affiliations:** 1 School of Physics and Electronic Information, Yantai University, Yantai 264005, China; muhanchao@s.ytu.edu.cn (H.M.); hpf_972@ytu.edu.cn (P.H.); 202400357024@s.ytu.edu.cn (J.C.); 202400357094@s.ytu.edu.cn (W.W.); 2 Shandong Data Open Innovation Application Laboratory of Smart Grid Advanced Technology, Yantai 264005, China

**Keywords:** mobile edge computing (MEC), reusable task, cooperative offloading, resource allocation, remaining energy levels

## Abstract

As an emerging paradigm for supporting computation-intensive and latency-sensitive services, mobile edge computing (MEC) faces significant challenges in terms of efficient resource utilization and intelligent task coordination among heterogeneous user equipment (UE), especially in dense MEC scenarios with severe interference. Generally, task similarity and cooperation opportunities among UE are usually ignored in existing studies when dealing with reusable tasks. In this paper, we investigate the problem of cooperative computation offloading and resource allocation for reusable tasks, with a focus on minimizing the energy consumption of UE while ensuring delay limits. The problem is formulated as an intractable mixed-integer nonlinear programming (MINLP) problem, and we design a similarity-based cooperative offloading and resource allocation (SCORA) algorithm to obtain a solution. Specifically, the proposed SCORA algorithm decomposes the original problem into three subproblems, i.e., task offloading, resource allocation, and power allocation, which are solved using a similarity-based matching offloading algorithm, a cooperative-based resources allocation algorithm, and a concave–convex procedure (CCCP)-based power allocation algorithm, respectively. Simulation results show that compared to the benchmark schemes, the SCORA scheme can reduce energy consumption by up to 51.52% while maintaining low latency. Moreover, the energy of UE with low remaining energy levels is largely saved.

## 1. Introduction

In recent years, the rapid advancement of wireless communications and internet of things (IoT) technologies has driven a surge in mobile user equipment (UE) and IoT devices. Concurrently, 5G mobile communication technology has reached full-scale commercialization, spurring the emergence of computation-intensive and latency-sensitive applications such as biometric recognition (face/fingerprint/iris), natural language processing, and interactive gaming [1]. These applications require significant amounts of both energy and computing power from UE. However, the constrained computing and battery capacities of UE often hinder efficient operation, adversely affecting the quality of experience (QoE) of UE. Moreover, reliable communication quality and low network latency are essential for these applications. Proposed by the European Telecommunications Standards Institute (ETSI), mobile edge computing (MEC) has emerged as an effective extension of traditional mobile cloud computing (MCC) [2]. In technical specification [3], support for edge computing by the 3rd Generation Partnership Project (3GPP) is described. MEC enables operator and third-party services to be hosted close to the UE’s access point so as to reduce end-to-end latency and transport network load. The fundamental principle of MEC involves processing data at the network edge through MEC servers deployed in proximity to UE. This architecture allows MEC servers to handle latency-sensitive and computation-intensive tasks locally, enabling elastic utilization of computational and storage resources. Consequently, MEC effectively addresses traditional cloud computing challenges including high latency, excessive energy consumption, and data security risks associated with long-distance data transmission.

In dense edge computing systems (DECS), which integrate ultra-dense network (UDN) and MEC, the growing UE population necessitates the adoption of loosely coupled reusable task design as a critical architectural solution. This design paradigm enables applications to be composed of modular tasks where input parameters and output results are decoupled from task code implementation, allowing different input combinations with the same task code to generate corresponding outputs [4]. For example, virtual reality (VR) and augmented reality (AR) applications require a large amount of real-time rendering and data processing, and multiple pieces of UE may need to handle the same rendering and data processing tasks at the same time within the same region, with only the UE’s own data being different. These tasks can be regarded as reusable tasks, and multiple pieces of UE can share the same rendering resources and processing results through cooperation. Moreover, there are three characteristic scenarios that demonstrate the potential of reusable tasks: (1) For connected vehicle ecosystems, traffic incidents during peak hours often trigger simultaneous requests from numerous UE items for identical real-time navigation data and updated traffic conditions [4]. (2) In a component-based multiplayer gaming environment, multiple players may frequently reuse the same game components [5]. (3) In industrial IoT deployments, massive IoT devices often generate reusable task requests in the same scenarios [6].

In this paper, taking the task similarity into consideration, we want to connect multiple pieces of UE with numerous identical reusable tasks to the same MEC server. In this way, during the processing phase of each task, only one UE device is required to offload the task code to the MEC server for computation. The remaining UE devices merely need to send the task input parameters and can share the corresponding task output results, aiming to reduce energy consumption. In addition, in order to improve the QoE of UE, our goal is to minimize the energy consumption of UE by jointly optimizing the task offloading decisions, subchannel and computing resource allocation, and transmission power allocation while considering the remaining energy of UE. Therefore, we propose a similarity-based cooperative offloading and resource allocation (SCORA) algorithm to achieve our objectives. The main contributions of this paper are as follows:

1. To address the cooperative offloading problem posed by reusable tasks in DECS, we formulate a joint optimization problem involving offloading decisions, subchannel assignments, computing resource allocation, and transmission power optimization to minimize the energy consumption of UE;
2. Since the formulated problem is a mixed-integer nonlinear programming problem (MINLP), to facilitate the solution of this problem, we decompose the problem into three subproblems and solve them separately. For the offloading subproblem, considering the task similarity between UE devices, a similarity-based matching offloading strategy is developed to solve it; then, a cooperative-based subchannel allocation strategy is proposed to solve the subchannel allocation subproblem, and finally, the UE devices’ transmission power is optimized using the concave–convex procedure (CCCP) method;
3. Simulation results show that our scheme performs better than existing ones. It can effectively reduce the average energy consumption and latency of user equipment. Moreover, it can save power for UE with low remaining energy levels, and it adapts well to different numbers of MEC servers and UE devices, providing a reliable solution for reusable task offloading and resource allocation in DECS.

The organization of this article is as follows. Section 2 describes the related work. Section 3 introduces the system model, including the network model, the task model, the communication model, the latency model and the energy consumption model, and then formulates the problem. In Section 4, we decompose the proposed problem into three sub-problems and solve these three sub-problems, respectively. Section 5 provides the related simulations. Finally, we conclude this article in Section 6.

## 2. Related Work

Contemporary academic studies have systematically addressed crucial technical aspects including offloading policy formulation and heterogeneous resource orchestration in the MEC system. In [7], the authors proposed a collaborative offloading scheme between MEC servers in order to alleviate network congestion and then used the deep Q-network approach to minimize the total execution time concerning deadline constraints. In [8], the authors added energy consumption constraints while constructing the goal of minimizing system latency. There are many studies aiming to minimize energy consumption [9,10,11]. In [9], the authors proposed an offloading strategy for the joint optimization of computing and communication resources to minimize energy consumption within the maximum tolerance time. In [10], the authors considered minimizing energy consumption while maximizing the number of tasks completed in a dynamical system, and they proposed a deep reinforcement learning scheme to jointly optimize the offloading decisions and the computational frequency allocation. In [11], the authors added service migration cost and task discarding penalties to the proposed objective of minimizing energy consumption in mobile IoT networks using energy harvesting, and they optimized the harvested energy, task allocation factor, central processing unit (CPU) frequency, transmission power, and association vector. In order to obtain a balance of latency and energy consumption, many studies focus on multi-objective optimization frameworks with approaches such as game theory and machine learning algorithms [12,13,14,15]. In [12], the authors proposed an improved quantum particle swarm algorithm to optimize task offloading decisions. In [13], considering the download delay of tasks, the authors proposed a three-stage multi-round combined offload scheduling mechanism and a joint resource allocation policy to solve the joint optimization problem of task offloading and heterogeneous resource allocation. In [14], the authors considered idle offsite servers and UE mobility, and they proposed a multilateral collaborative computation offloading model and an improved genetic algorithm. In [15], the authors formulated the system utility as an integrated function of computing service costs, task execution time, and energy consumption and then optimized the joint optimization of offloading decisions, transmission power, computing resource allocation, and computational service costs through a two-tier bargaining-based task offloading and a collaborative computing incentive mechanism.

In the aforementioned studies, although the increased number of MEC servers has proven to be effective in assisting task processing, the quantity of MEC servers typically remains limited. In DECS, the proliferation of MEC servers enhances opportunities for task offloading and collaborative processing among UE devices with rapidly increasing density. However, it also brings some challenges, such as the complexity of task offloading decisions, multidimensional resource allocation, and intricate service migration and caching placement issues. Many studies have endeavored to address the task offloading problems [16,17,18]. In [16], in order to minimize the long-term average task latency of all UE, the authors developed a novel calibrated contextual bandit learning algorithm to enable UE devices to predict the task offloading decisions of the rest of the UE in order to independently decide their own offloading decisions. In [17], the authors designed an online task offloading deep reinforcement learning algorithm: the asynchronous advantage actor–critic. This framework operates independently of real-time channel state information and BS computational power, achieving the dual objectives of strict adherence to energy budget constraints and systematic minimization of task completion latency. In [18], the authors proposed a contextual sleeping bandit learning algorithm (CSBL) with Lyapunov optimization to minimize long-term task delay under price constraints, and they extended it to multi-server scenarios as CSBL-M to address exponential action space growth in task offloading.

In DECS, the proliferation of UE has given rise to pressing resource allocation challenges. Therefore, many of recent studies have focused on addressing computational offloading and resource allocation problems [19,20,21,22]. In [19], the authors proposed a deep reinforcement learning-based scheduling algorithm that utilizes deep deterministic policy gradient and behavioral critique networks to solve the task scheduling and resource scheduling problems, aiming to minimize the task latency for all UE devices. In [20], in order to minimize the system energy consumption, the authors used the improved artificial fish swarm algorithm and the improved particle swarm optimization (PSO) algorithm to jointly optimize the computation offloading decision, the task offloading ratio, and the allocation of communication and computation resources. In [21], the authors jointly optimized the problems of task offloading, BS selection, and resource scheduling, which is addressed by a Newton-interior point method-based resource allocation algorithm and a genetic algorithm-based scheduling method, achieving the minimization of the weighted sum of system delay and energy consumption. In [22], taking the uncertainties in UE mobility and resource constraints into consideration, the authors proposed a distributed delay-constrained computation offloading framework that incorporates Lyapunov-based game-theoretic optimization and multi-stage stochastic programming to achieve adaptive task offloading and computational capacity management. In addition, extensive studies exist on dynamic service migration and caching placement mechanisms [23,24]. An energy-efficient online algorithm based on Lyapunov and PSO was developed to solve the task migration problem in order to reduce energy consumption while considering the interference and mobility of UE in [23]. In [24], the authors proposed a two-timescale hierarchical multi-agent deep reinforcement learning (HMDRL)-based scheme for the joint optimization of cooperative service caching, computation offloading, and resource allocation to minimize weighted energy consumption across energy-harvesting (EH)-powered mobile UE devices and small base stations (SBSs). Although these studies addressed many problems in DECS, they failed to consider the homogeneity of tasks between UE devices and ignored cooperation opportunities brought about by the dense deployment of UE devices and MEC servers.

In the processing of reusable tasks, cooperative offloading realizes resource sharing via task decomposition, resource allocation, and UE cooperation to boost efficiency and reduce energy consumption. To fully exploit the computing capacity of a multi-server system, collaborative offloading among multiple edge servers is necessary [25]. Scenarios involving reusable tasks are considered in [4,5,6,26]. In [4], by applying coalitional game theory, the authors formulated a cooperative offloading process for reusable tasks as a coalitional game to maximize cost savings. In [5], the authors developed a 0–1 integer nonlinear programming problem to minimize the total energy cost on the player’s side under a delay constraint in a component-based multiplayer game scenario. In [6], the authors incorporated drones to assist with the offloading of reusable tasks in an MEC environment. By jointly optimizing the UE offloading policy, UE transmission power, server allocation on the unmanned aerial vehicle (UAV), the computation frequency of the UE and the UAV server, and the UAV flight trajectory, a system model was constructed to minimize the system average total energy consumption under time delay constraints. In [26], the authors formulated the joint offload optimization problem with the aim of minimizing the long-term average task execution cost, taking into account transmission collaboration, shared wireless bandwidth, and varying task queues in UE devices and MEC servers. The above research on cooperative task offloading does not take into account the types of tasks in the scenario, which is important for the completion of cooperative offloading.

## 3. System Model and Problem Formulation

In this section, we will provide a detailed introduction to the system model, task model, delay model, and energy consumption model and present our optimization problem.

### 3.1. System Model

**Figure 1:**
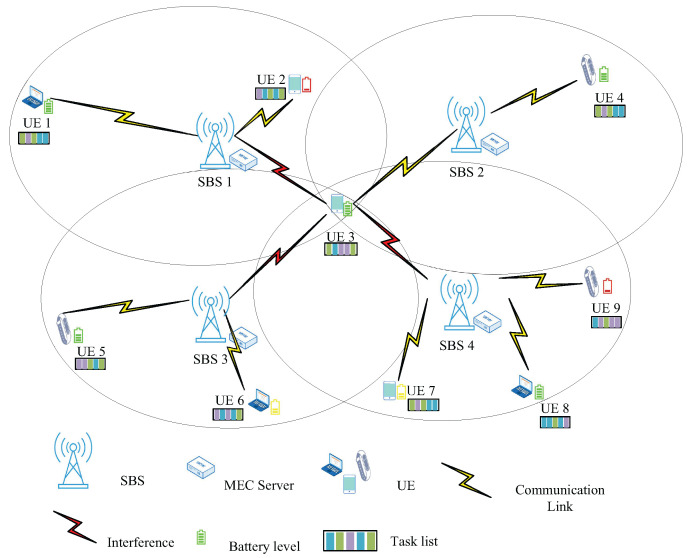
System model.

As shown in Figure 1, there are $M\;=\;\left\{1,2,\ldots ,M\right\}$ SBSs in our system, each equipped with an MEC server, using orthogonal frequency division multiple access technology (OFDMA). There are $N\;=\;\left\{1,2,\ldots ,N\right\}$ UE devices in the coverage area, each with its own remaining energy level and task list. The MEC server connected to the SBS is powered by cable, so the energy consumption of its processing tasks is not considered [27]. The energy consumption generated by the hardware of the UE’s own circuitry is chosen to be ignored [28]. The download latency of task results is usually ignored due to the small size of the output results and the generally high rate of the downlink [29,30]. When UE devices offload their tasks by occupying the same subchannels, the interference cannot be neglected [31]. For instance, as shown in Figure 1, when UE 3 offloads its computational tasks to SBS 2 via a subchannel, it generates interference to other SBSs serving UE devices operating on the same subchannel. The main symbols and their definitions are summarized in Table 1.

### 3.2. Task Model

**Figure 2:**
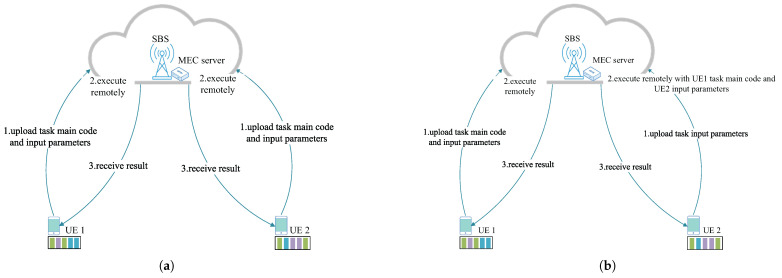
An example of reusable task offloading. (**a**) Non-cooperative offloading strategy. (**b**) Cooperative offloading strategy.

In each time slot, each UE device generates a type of task. Let *j* denote the computational task type and each UE device maintain a task list that stores the tasks. Figure 2 shows an example of reusable offloading. In Figure 2a, with the existing offloading method, even though the current reusable tasks of UE 1 and UE 2 are the same, all the task codes are offloaded separately to the MEC server for processing. However, as shown in Figure 2b, when UE 1 and UE 2 choose to perform cooperative offloading, if UE 1 offloads the task body code and its own input parameters, then UE 2 only needs to offload its own input parameters, which greatly reduces the energy consumption of UE 2. Therefore, we focus on the cooperative offloading. In order to save energy, UE devices will offload their computational tasks to MEC servers for processing due to limited computational power [32]. The reusable tasks in this paper are similar to [5], which does not consider the divisibility. Let $S_{nj}=\left(L_{nj},L_{nj,main},L_{nj,in},C_{nj},C_{nj,main},C_{nj,in},T_{nj}^{\max}\right)$ represent the attributes of the UE *n* unloading task *j* [33], where $L_{nj}$, $L_{nj,main}$, and $L_{nj,in}$ are the total task size, the task main code size, and the task input parameters size for UE *n*, respectively. $C_{nj}$, $C_{nj,main}$ and $C_{nj,in}$ are the number of CPU cycles required to complete the total task, the number of CPU cycles required to complete the task main code, and the number of CPU cycles required to complete the task input parameters, respectively. $T_{nj}^{\max}$ is the latency requirement for the UE *n* to complete task *j*.

### 3.3. Communication Model

Let $a_{mn}$ be a variable indicating task offloading. When UE *n* offloads its tasks to the MEC server *m* for processing, $a_{mn}=1$; otherwise, $a_{mn}=0$. Each MEC server divides bandwidth into *K* subchannels, with a set of $K\;=\;\left\{1,2,\ldots ,K\right\}$ subchannels and a bandwidth of $B_{0}$ for each subchannel. The accessed UE devices offload tasks in an orthogonal manner. Let $b_{mnk}$ denote the variable of subchannel allocation. When subchannel *k* is occupied by UE *n* to offload tasks to MEC server *m*, $b_{mnk}=1$; otherwise, $b_{mnk}=0$. Since OFDMA technology is used, there is no interference between UE devices connected to the same MEC server, but there is co-tier interference between different MEC servers, i.e., UE devices occupying the same subchannel have influence on each other [34]. According to the Shannon formula, the uplink data rate of UE *n* transmitting to MEC server *m* via subchannel *k* can be calculated as

(1) $$R_{n}=\sum_{m=1}^{M}\sum_{k=1}^{K}a_{mn}B_{0}log_{2}\left(1+\frac{b_{mnk}P_{nk}h_{mnk}}{\sigma^{2}+I_{mk}}\right),$$

where $I_{mk}=\sum_{i=1,i\neq n}^{N}a_{mi}b_{mik}P_{ik}h_{mik}$ is the co-tier interference to MEC server *m* on subchannel *k*, $P_{nk}$ is the transmission power of UE *n* on subchannel *k*, $h_{mnk}$ is the gain from UE *n* to MEC server *m* on subchannel *k*, and $\sigma^{2}$ is the Gaussian white noise power.

### 3.4. Latency Model

In the cooperative scheme, one piece of UE is selected from the UE devices with reusable tasks within the same MEC servers to offload the main task code and its own task input parameters to the MEC server. The remaining UE devices only need to send their own task input parameters to the MEC server. Then, the MEC server will calculate each task and send the results of each task back to each piece of UE. If UE *n* accesses MEC server *m* and acts as the UE for offloading the main part and input parameters of the task, then the size of its offloaded task can be given by

(2) $$L_{nj}=L_{nj,main}+L_{nj,in}.$$

The number of CPU cycles required to complete the entire task can be given by

(3) $$C_{nj}=C_{nj,main}+C_{nj,in}.$$

The transmission latency of UE *n* can be calculated by

(4) $$T_{nj}^{t}=\sum_{m=1}^{M}a_{mn}\frac{L_{nj}}{R_{n}}=\sum_{m=1}^{M}a_{mn}\frac{L_{nj,main}+L_{nj,in}}{R_{n}}.$$

The computational latency of the MEC server *m* for the task computation of UE *n* can be expressed as

(5) $$T_{nj}^{s}=\sum_{m=1}^{M}a_{mn}\frac{C_{nj}}{f_{n}}=\sum_{m=1}^{M}a_{mn}\frac{C_{nj,main}+C_{nj,in}}{f_{n}},$$

where $f_{n}$ is a computing resource allocated by the MEC server *m* to the accessed UE *n*.

If UE *n* is used as a UE device that offloads only the task input parameters, then its offload task size should be $L_{nj}=L_{nj,in}$, and the number of CPU cycles required to complete the entire task should be $C_{nj}=C_{nj,in}$.

The transmission latency of UE *n* is calculated as follows:

(6) $$T_{nj}^{t}=\sum_{m=1}^{M}a_{mn}\frac{L_{nj}}{R_{n}}=\sum_{m=1}^{M}a_{mn}\frac{L_{nj,in}}{R_{n}}.$$

Since the task can only start to be executed after all the task data have been uploaded to the MEC server, the UE devices that send the task input parameters need to wait until the UE item that sends the main part of the task has completed the offloading before they can start the task execution. If the transmission delay of UE *n* is less than that of the cooperative UE *i* which offloads the main code of task *j*, then it needs to wait until the offload of cooperative UE *i* is completed before the task calculation can be carried out. If it is greater than the transmission delay of the cooperative UE *i*, there is no need to wait; that is, $T_{nj}^{t}=\max\left\{T_{nj}^{t},T_{ij}^{t}\right\}$, where i≠n, i∈N, $T_{ij}^{t}=\sum_{m=1}^{M}a_{mi}\frac{L_{ij}}{R_{i}}$. Moreover, since the power usage of the UE during the waiting period is very small, we choose to ignore the energy consumption during the waiting process.

The computational latency of the MEC server *m* for task computation $T_{nj}^{s}$ is (5). Therefore, the total latency of UE *n* can be expressed as

(7) $$T_{nj}=T_{nj}^{t}+T_{nj}^{s}=\sum_{m=1}^{M}a_{mn}\left(\frac{L_{nj}}{R_{n}}+\frac{C_{nj}}{f_{n}}\right).$$

### 3.5. Energy Consumption Model

When UE *n* is used as the UE device that sends the task main code and task input parameters, the transmission energy consumption of UE *n* can be expressed as

(8) $$E_{nj}^{t}=\sum_{k=1}^{K}P_{nk}T_{nj}^{t}=\sum_{k=1}^{K}\sum_{m=1}^{M}a_{mn}P_{nk}\frac{L_{nj,main}+L_{nj,in}}{R_{n}},$$

where $P_{nk}$ is the power of UE *n*.

When UE *n* is used as the UE device that only sends its task input parameters, the transmission energy consumption of UE *n* can be calculated by

(9) $$E_{nj}^{t}=\sum_{k=1}^{K}P_{nk}T_{nj}^{t}=\sum_{k=1}^{K}\sum_{m=1}^{M}a_{mn}P_{nk}\frac{L_{nj,in}}{R_{n}}.$$

Finally, the total energy consumption of UE *n* is calculated as follows:

(10) $$E_{nj}=E_{nj}^{t}=\sum_{k=1}^{K}\sum_{m=1}^{M}a_{mn}P_{nk}\frac{L_{nj}}{R_{n}}=\sum_{k=1}^{K}\sum_{m=1}^{M}\frac{a_{mn}P_{nk}L_{nj}}{\sum_{m=1}^{M}\sum_{k=1}^{K}a_{mn}B_{0}log_{2}\left(1+\frac{b_{mnk}P_{nk}h_{mnk}}{\sigma^{2}+I_{mk}}\right)}.$$

### 3.6. Problem Formulation

In this paper, considering the similarity task, we jointly optimize task offloading policy, the allocation of communication and computing resources, and the transmission power of UE devices to minimize the average total energy consumption of the UE devices. The problem is formulated as follows:

(11) $$\begin{array}{cc} \; & \min_{a_{mn},b_{mnk},f_{n},P_{nk}}\frac{1}{N}\sum_{n=1}^{N}E_{nj}\\ \; & s.t.C1:\sum_{m=1}^{M}a_{mn}=1,\forall n\in N\\ \; & \;C2:\sum_{m=1}^{M}\sum_{k=1}^{K}b_{mnk}=1,\forall n\in N\\ \; & \;C3:a_{mn},b_{mnk}\in \left\{0,1\right\}\\ \; & \;C4:P_{nk}\leq P_{nk}^{\max},\forall n\in N,\forall k\in K\\ \; & \;C5:\sum_{k=1}^{K}P_{nk}\leq P_{n}^{\max},\forall n\in N\\ \; & \;C6:T_{nj}\leq T_{nj}^{\max},\forall n\in N, \end{array}$$

where $P_{nk}^{\max}$ and $P_{n}^{\max}$ are the max transmission power on each subchannel and on each MEC server for UE *n*.

Constraint C1 indicates that each UE device can select only one MEC server for computation offloading. Constraint C2 indicates that each piece of UE is assigned only one subchannel. Constraint C3 indicates that both task offloading and subchannel assignment are binary. Constraints C4 and C5 give the transmission power limit on each subchannel and on each MEC server, respectively. Constraint C6 is the maximum delay requirement for UE *n* to complete the task. Since problem (11) is an MINLP problem, obtaining the optimal solution is challenging.

## 4. Similarity-Based Cooperative Offloading and Resource Allocation Algorithm

**Figure 3:**
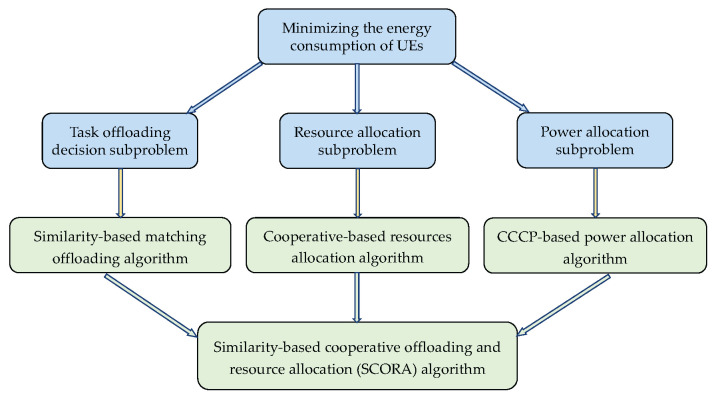
Relationship between subproblems and the corresponding algorithms.

In this section, we propose the SCORA algorithm to solve the formulated problem (11). We divide it into three interdependent subproblems: (1) a task offloading decision subproblem, (2) a resource allocation subproblem, and (3) a power allocation subproblem. In the SCORA algorithm, the similarity-based matching offloading algorithm is proposed to solve the task offloading decision subproblem, and the cooperation-based resource allocation algorithm is designed to tackle the resource allocation problem, leveraging task similarity among UE devices. Finally, the non-convex power allocation optimization is systematically addressed through the CCCP-based power allocation algorithm, enabling efficient energy consumption minimization. Figure 3 illustrates the original problem and the decomposed subproblems and corresponding algorithms.

### 4.1. Similarity-Based Matching Offloading Algorithm

Given that each UE offloads its task to exactly one MEC server, while each MEC server is capable of serving multiple UE devices, it is suitable for this problem to be solved by applying many-to-one matching theory. Initially, the matching multinomial group $\left(P_{M},\psi ,\Phi \right)$ is defined as follows:

1. $P_{M}=\left\{M,N\right\}$ are two sets of unrelated collections. In this paper, M is the set of MEC servers that executes tasks, and N is the set of UE devices that offloads tasks to MEC servers.
2. $\psi =\left(PL_{m},L_{n}\right)$ represents a list of preferences for MEC servers and UE. Each MEC server m∈M maintains a preference list $PL_{m}$ in which the preferences for UE devices are sorted in descending order, that is, $PL_{m}=\left\{n,n{≻}_{m}n^{\prime }\right\}$, which means that the MEC server *m* prefers UE *n* to $n^{\prime }$. Each UE device maintains a preference list $PL_{n}=\left\{m,m{≻}_{n}m^{\prime }\right\}$ for MEC servers in descending order as well.
3. Φ⊆M×N represents the matching between MEC servers and UE. Each UE device can be matched with at most one MEC server, that is, $\Phi \left(n\right)\in M$ and $\left|\Phi \left(n\right)\right|\leq 1$, where $\left|\Phi \left(\cdot \right)\right|$ is the cardinality of the matching result $\Phi \left(\cdot \right)$. In addition, each MEC server can be matched with multiple UE devices, that is, $\Phi \left(m\right)\in N$ and $\left|\Phi \left(m\right)\right|\leq A_{m}$, where $A_{m}$ is maximum number of access UE devices for the MEC server *m*.

Subsequently, the definition of the considered many-to-one matching can be described as follows:

**Definition** **1.** *Given the UE set N and the MEC server set M, the matching game Φ:N→M for computation offloading is defined as a many-to-one function, such that*

1. *$\Phi \left(n\right)\in M$ and $\left|\Phi \left(n\right)\right|\leq 1,\forall n\in N$;*
2. *$\Phi \left(m\right)\in N$ and $\left|\Phi \left(m\right)\right|\leq K,\forall m\in M$;*
3. *$n=\Phi \left(m\right)\Leftrightarrow m=\Phi \left(n\right),\forall n\in N,\forall m\in M$.*

During the computation offloading process, each UE device is willing to associate with the MEC server that has the best channel conditions to obtain a high offloading rate. Therefore, a preference function of UE *n* for each MEC server is established:

(12) $$\psi_{n}\left(m\right)=h_{mnk}.$$

Each UE device can calculate preferences for the MEC servers and generate a preference list based on the above equation.

In an effort to effectively pair more UE devices that have identical reusable tasks with the same MEC server, the similarity score is introduced as a metric. For example, the similarity score $G\left(i,n\right)$ between UE *i* and UE *n* can be calculated in the following way: First, the number of similar tasks in the task lists of UE *i* and UE *n* is counted, and then $G\left(i,n\right)$ can be calculated as a ratio of the number of similar tasks to the total number of tasks of UE *i*. Before starting the matching algorithm, we calculate the similarity scores among UE devices. These scores are incorporated into the preference function of MEC servers for UE. In this way, they influence the preferences of UE when choosing the same MEC server, thereby enabling the pairing of more UE devices with identical reusable tasks to the same MEC server. The preference function of the MEC server *m* for each UE device can reflect that the MEC server has a higher preference for UE with good channel conditions and low energy consumption, which can prioritize the tasks of UE devices with low remaining energy levels to save the energy, and the addition of similarity scoring can match more UE devices with the same tasks to the same MEC server to achieve more energy savings for UE. The preference function of MEC server *m* for each UE device can be calculated as

(13) $$\psi_{m}\left(n\right)=\left[\left(1-\beta \right)\frac{h_{mnk}}{{\bar{\varepsilon }}_{n}}+\beta G\left(i,n\right)\right],$$

where ${\bar{\varepsilon }}_{n}$ is the remaining energy level of UE *n*.

Algorithm 1 summarizes the steps of similarity-based matching offloading strategy. First, all UE devices and MEC servers are not matched. Therefore, the unmatched UE set $N_{u}$ and the unmatched MEC server set $M_{u}$ are initialized as all UE and all MEC servers, respectively. Calculate the similarity score $G\left(i,n\right)$ between UE *i* and UE *n*. In the matching process, every unmatched UE device builds its preference list based on (12) and sends an offloading request to the first MEC server on the preference list. Subsequently, each unmatched MEC server calculates $m\in M_{u}$, which is the number of offloading requests received, and marks UE devices that sent the offloading requests by the set $M_{C_{m}}$. If $C_{m}$ is not more than the maximum number of access UE devices for MEC server *m* $A_{m}$, then all UE in $M_{C_{m}}$ is allowed to communicate with the MEC server *m* for task offloading. Otherwise, the unmatched MEC server *m* establishes its preference list based on (13). The first $A_{m}$ UE devices in $M_{C_{m}}$ are allowed to offload their tasks, and the offloading requests of the other UE in $M_{C_{m}}$ are rejected. Then, the unmatched UE set $N_{u}$ and the unmatched MEC server set $M_{u}$ are updated. The matching process is not finished until all UE devices and MEC servers are matched. Finally, the offloading indicator can be obtained from the matching result $A^{*}$. Following the same method as in [31,35], we can prove that the proposed matching algorithm is stable.
**Algorithm 1** Similarity-based matching offloading algorithm.**Input:** The maximum latency requirement $T_{nj}^{\max}$ for all UE to complete the task.**Output:** Stable matching result $A^{*}$.  1:Initialize the unmatched MEC server set $M_{u}=\left\{1,2,\ldots ,M\right\}$, and the unmatched UE set $N_{u}=\left\{1,2,\ldots ,N\right\}$. Calculate the similarity score $G\left(i,n\right)$ between UE $i\in N_{u}$ and UE $n\in N_{u}$.  2:**while**$N_{u}\neq \emptyset$ **do**  3:       **for** all unmatched UE $n\in N_{u}$ **do**  4:            UE *n* builds its preference list $PL_{n}$ based on (12) in a descending order;  5:            UE *n* sends an offloading request to the first MEC server in $PL_{n}$;  6:       **end for**  7:       **while** $M_{u}\neq \emptyset$ **do**  8:             **for** all unmatched MEC server $m\in M_{u}$ **do**  9:                   Count the number of requests received by MEC server *m* as $C_{m}$ and denote                    the set of these UE devices as $M_{C_{m}}$;10:                   **if** $C_{m}\leq A_{m}$ **then**11:                       MEC server *m* allows these $C_{m}$ UE devices to offload their tasks;12:                       Remove MEC server *m* from $M_{u}$ and $PL_{n}$, and remove these $C_{m}$ UE                       devices from $N_{u}$;13:                   **end if**14:                   **if** $C_{m}>A_{m}$ **then**15:                        MEC server *m* updates its preference list $PL_{m}$ in a descending order                         according to (13) and allows the first *K* UE devices in $M_{C_{m}}$ to offload                         their tasks;16:                        Remove MEC server *m* from $M_{u}$ and $PL_{n}$, and remove these $C_{m}$ UE                         devices from $N_{u}$;17:                   **end if**18:             **end for**19:       **end while**20:       For all UE devices n∈N matched with MEC server *m*, $a_{mn}=1$;21:**end while**

### 4.2. Cooperation-Based Resource Allocation Algorithm

To better achieve the purpose of cooperative offloading, we adopt the agglomerative clustering algorithm in the hierarchical clustering algorithm to form clusters of UE devices with the same type of reusable tasks in the current time slot. The agglomerative hierarchical clustering algorithm divides data points into clusters by calculating the distance between each cluster. In this paper, data points are defined as clusters formed by UE, and the total number of clusters is $D\;=\;\left\{1,2,\ldots ,D\right\}$. $N_{m}$ denotes the set of $A_{m}$ UE devices connected to MEC server *m*. $D_{m}$ represents the cluster formed by UE $i\in N_{m}$. The distance is defined as the task similarity between UE devices. We adopt the merging rule based on the shortest distance and use the task types $g_{i,j}$ and $g_{n,j}$ of the current time of UE *i* and UE *n* to calculate the distance $l_{i,n}$. The shortest distance among all clusters is set as $l_{\min}$, and the stopping condition is $l_{\max}$. If the task types $g_{i,j}$ and $g_{n,j}$ of UE *i* and UE *n* are the same, then $l_{i,n}=l_{\min}$. When $l_{\min}<l_{\max}$, the clusters $d_{i}$ and $d_{n}$ where UE *i* and UE *n* are located will be merged into $d_{in}$. Here, we introduce the cooperation-based resource allocation algorithm, as shown in Algorithm 2.
**Algorithm 2** Cooperation-based resource allocation algorithm.**Input:** The Stable matching result $A^{*}$.**Output:** The subchannel allocation result $Q^{*}$ and computing resources allocation result $F^{*}$.  1:**for** all MEC servers m∈M **do**  2:      **for** all UE devices $i\in N_{m}$ **do**  3:            If the task types $g_{i,j}$ and $g_{n,j}$ of UE *i* and UE *n* are the same, then $l_{i,n}=l_{\min}$;  4:             **while** $l_{\min}<l_{\max}$ **do**  5:                   The clusters $d_{i}$ and $d_{n}$ where UE *i* and UE *n* are located will merge into $d_{in}$;  6:                   Compare the task types $g_{in,j}$ of the $d_{in}$ with task types $g_{c,j}$ of all remaining                   clusters $d_{c},c\in N_{m},c\neq i,n$;  7:                   Remove UE *n* from $N_{m}$;  8:             **end while**  9:      **end for**10:      **for** all clusters $d_{i}\in D_{m}$ **do**11:            Allocate resources to cluster $d_{i}$ according to (14) and (18);12:            Select the cluster head $d_{i,h}$ according to (15);13:            Allocate resources to cluster head $d_{i,h}$ according to (16) and (19);14:            Allocate resources to cluster member $d_{i,o}$ according to (17) and (20);15:      **end for**16:**end for**

Each MEC server allocates the subchannels to each cluster according to the ratio of the number of UE devices. The number of subchannels allocated to cluster $d_{i}$ can be calculated by

(14) $$Q_{d}=K\times \frac{S_{d_{i}}}{\sum_{i=1}^{D}S_{d_{i}}},$$

where *K* is the number of subchannels in the system, and $S_{d_{i}}$ is the number of UE devices in cluster $d_{i}$.

All of the UE devices $n\in d_{i}$ are sorted based on the product of channel gain and residual energy, and the first piece of UE is selected as the cluster head $d_{i,h}$. The sorting formula is as follows:

(15) $$\psi_{d_{i}}\left(n\right)=h_{mnk}{\bar{\varepsilon }}_{n}.$$

In this way, the selected cluster head a the piece of UE with both high channel gain and remaining energy, which is beneficial to reducing the total energy consumption and delaying and reducing the energy consumption for UE with low remaining energy levels.

This strategy allocates subchannels to the cluster head and the remaining UE according to the ratio of the total task size sent by the cluster head and the total task size sent by other UE in the cluster. The number of subchannels allocated to the cluster head $d_{i,h}$ of cluster $d_{i}$ can be calculated by

(16) $$Q_{d_{i,h}}=Q_{d}\times \frac{L_{d_{i},main}+L_{d_{i,h},in}}{L_{d_{i},main}+\sum_{s=1}^{S_{d_{i}}}L_{d_{i,s},in}},$$

where $L_{d_{i},main}$ is the main code of the task to be sent by all UE in cluster $d_{i}$, $L_{d_{i,h},in}$ is the task input parameter of the cluster head $d_{i,h}$, and $\sum_{s=1}^{S_{d_{i}}}L_{d_{i,s},in}$ is the task input parameter of all UE in cluster $d_{i}$.

The number of subchannels $Q_{d_{i,o}}$ allocated to the cluster member UE $d_{i,o}$ in cluster $d_{i}$ can be expressed as

(17) $$Q_{d_{i,o}}=Q_{d_{i}}\times \frac{L_{d_{i,o},in}}{L_{d_{i},main}+\sum_{s=1}^{S_{d_{i}}}L_{d_{i,s},in}},$$

where $L_{d_{i,o},in}$ is the task input parameter of the cluster member UE $d_{i,o}$ in cluster $d_{i}$.

The number of computing resources allocated to cluster $d_{i}$ can be calculated by

(18) $$F_{d}=K\times \frac{S_{d_{i}}}{\sum_{i=1}^{D}S_{d_{i}}}.$$

The number of computing resources allocated to the cluster head $d_{i,h}$ of cluster $d_{i}$ can be calculated by

(19) $$F_{d_{i,h}}=F_{d}\times \frac{L_{d_{i},main}+L_{d_{i,h},in}}{L_{d_{i},main}+\sum_{s=1}^{S_{d_{i}}}L_{d_{i,s},in}}.$$

The number of computing resources allocated to the cluster member UE $d_{i,o}$ in cluster $d_{i}$ can be expressed as

(20) $$F_{d_{i,o}}=F_{d_{i}}\times \frac{L_{d_{i,o},in}}{L_{d_{i},main}+\sum_{s=1}^{S_{d_{i}}}L_{d_{i,s},in}}.$$

### 4.3. CCCP-Based Power Allocation Algorithm

After completing the computation offloading and resource allocation, the problem becomes

(21) $$\begin{array}{cc} \; & \min_{P_{nk}}\frac{1}{N}\sum_{n=1}^{N}\frac{\sum_{k=1}^{K}P_{nk}L_{nj}}{\sum_{k=1}^{K}B_{0}\log_{2}\left(1+\frac{P_{nk}h_{mnk}}{\sigma^{2}+I_{mk}}\right)}\\ \; & s.t.C4.C5.C6. \end{array}$$

To better accomplish the power optimization, the problem is rewritten as follows:

(22) $$\begin{array}{cc} \; & \min_{P_{nk}}\frac{1}{N}\sum_{n=1}^{N}\sum_{k=1}^{K}\varphi_{nk}\\ \; & s.t.C4,C5,C6,\\ \; & C7:\frac{P_{nk}L_{nj}}{B_{0}log_{2}\left(1+\frac{P_{nk}h_{mnk}}{\sigma^{2}+I_{mk}}\right)}\leq \varphi_{nk}. \end{array}$$

Theorem 1 is introduced to make the above equation tractable.

**Theorem** **1.** If $\left(P^{*},\varphi^{*}\right)$ is the optimal solution of the problem, then there exist $\eta_{nk}^{*}$, n=1,…,N, k=1,…,K when $\eta =\eta^{*}$ and $\varphi =\varphi^{*}$, such that $P^{*}$ is a solution to the following problem:

(23) $$\begin{array}{cc} \; & \min_{P_{nk}}\frac{1}{N}\sum_{n=1}^{N}\sum_{k=1}^{K}\eta_{nk}\left[\varphi_{nk}B_{0}\log_{2}\left(1+\frac{P_{nk}h_{mnk}}{\sigma^{2}+I_{mk}}\right)-P_{nk}L_{nj}\right]\\ \; & s.t.C4,C5,C6,C7. \end{array}$$

and when $\eta =\eta^{*}$ and $\varphi =\varphi^{*}$, $P^{*}$ also satisfies the following equations:

(24) $$\varphi_{nk}=\frac{P_{nk}L_{nj}}{R_{n}},\eta_{nk}=\frac{1}{R_{n}},\forall n\in N,\forall k\in K.$$

According to Theorem 1, the optimal solution of problem (21) can be obtained by solving (23). If the solution of (23) is unique, then the solution of (23) is the optimal solution of problem (22). Next, we will solve (23).

After rearranging the objective function in (24), we obtain

(25) $$U\left(P\right)=U_{cave1}\left(P\right)-U_{cave2}\left(P\right),$$

where the functions on the right side of the equation are

$$\begin{array}{c} U_{cave1}\left(P\right)=\frac{1}{N}\sum_{n=1}^{N}\sum_{k=1}^{K}\eta_{nk}\varphi_{nk}B_{0}\log_{2}\left(\sum_{t=1}^{N}P_{tk}h_{mtk}+\sigma^{2}\right) \end{array}$$

and $U_{cave2}\left(P\right)=\frac{1}{N}\sum_{n=1}^{N}\sum_{k=1}^{K}\eta_{nk}\varphi_{nk}B_{0}\log_{2}\left(\sigma^{2}+I_{mk}\right)+\frac{1}{N}\sum_{n=1}^{N}\eta_{nk}P_{nk}L_{nj}$.

In addition, the non-convex constraint condition C6 can be transformed into its equivalent convex linear form $C6^{\prime }$ through mathematical operations:

(26) $$C6^{\prime }:P_{nk}h_{mnk}+\left[1-2^{\frac{L_{nj}}{B_{0}\left(T_{nj}^{\max}-\frac{C_{nj}}{f_{n}}\right)}}\right]\left(\sigma^{2}+I_{mk}\right)\geq 0.$$

Therefore, problem (23) is equivalent to

(27) $$\begin{array}{cc} \; & \min_{P_{nk}}U\left(P\right)\\ \; & s.t.C4,C5,C6^{\prime },C7. \end{array}$$

The solution of problem (21) can be obtained by solving (27). Since $U_{cave1}\left(P\right)$ and $U_{cave2}\left(P\right)$ are both concave functions with respect to P, problem (24) has a difference-of-convex structure. Moreover, since $U_{cave2}\left(P\right)$ is differentiable, the CCCP algorithm can be used to solve problem (27). The main idea of the CCCP algorithm is to transform the convex part of U(P), that is, $-U_{cave2}\left(P\right)$, into a linear form through iteration. By introducing Theorem 2, the above problem can be solved.

**Theorem** **2.** *Problem (27) can be solved by solving the following sequential convex programming problem:*

(28) $$P^{\left(l+1\right)}=\underset{P_{nk}}{\arg\min}\left\{U_{cave1}\left(P\right)-P^{T}\times \nabla U_{cave2}\left(P^{\left(l\right)}\right)\right\},$$

*where $P^{T}$ is the transpose of P. $\nabla U_{cave2}\left(P^{\left(l\right)}\right)=\left[\nabla_{1}^{\left(l\right)},\nabla_{2}^{\left(l\right)},\ldots ,\nabla_{K}^{\left(l\right)}\right]$ represents the gradient of $U_{cave2}\left(P\right)$ at $P^{\left(l\right)}$ and $\nabla_{k}^{\left(l\right)}=\sum_{i=1,i\neq n}^{N}\frac{\eta_{ik}\varphi_{ik}B_{0}h_{mik}/\ln2}{\sum_{t=1,t\neq i}^{N}P_{tk}h_{mtk}+\sigma^{2}}+\eta_{nk}L_{nj}\overset{\Delta }{=}D_{nk}\left(\eta_{nk},\varphi_{nk}\right)$.*

Since (28) is a convex optimization problem, classical convex optimization algorithms can be used to solve the problem. The Lagrangian function corresponding to the construction of (28) is

(29) $$\begin{array}{cc} \; & L\left(P,\gamma ,\lambda \right)=\frac{1}{N}\sum_{n=1}^{N}\sum_{k=1}^{K}\eta_{nk}\varphi_{nk}B_{0}\log_{2}\left(I_{mk}+\sigma^{2}+P_{nk}h_{mnk}\right)\\ \; & -\frac{1}{N}\sum_{n=1}^{N}\sum_{k=1}^{K}P_{nk}D_{nk}\left(\eta_{nk},\varphi_{nk}\right)\\ \; & +\frac{1}{N}\sum_{n=1}^{N}\sum_{k=1}^{K}\gamma_{nk}\left[P_{nk}h_{mnk}+\left(1-2^{\frac{L_{nj}}{B_{0}\left(T_{nj}^{\max}-\frac{C_{nj}}{f_{n}}\right)}}\right)\left(\sum_{i=1,i\neq n}^{N}P_{ik}h_{mik}+\sigma^{2}\right)\right]\\ \; & -\frac{1}{N}\sum_{n=1}^{N}\lambda_{n}\left(\sum_{k=1}^{K}P_{nk}-P_{n}^{\max},\right) \end{array}$$

where γ and λ are Lagrange multipliers. Let $𝜕L\left(P,\gamma ,\lambda \right)/𝜕P_{nk}=0$ denote the Lagrange function, that is,

(30) $$\frac{B_{0}\eta_{nk}\varphi_{nk}}{\ln2}\times \frac{h_{mnk}}{I_{mk}+\sigma^{2}+P_{nk}h_{mnk}}-\left[D_{nk}\left(\eta_{nk},\varphi_{nk}\right)+\lambda_{n}\right]+\gamma_{nk}h_{mnk}+\sum_{i=1,i\neq n}^{N}\gamma_{ik}h_{mik}\left[1-2^{\frac{L_{nj}}{B_{0}\left(T_{nj}^{\max}-\frac{C_{nj}}{f_{n}}\right)}}\right]=0.$$

The optimum power is obtained as

(31) $$P_{nk}^{*}={\left[\frac{B_{n}\eta_{nk}\varphi_{nk}/\ln2}{D_{nk}\left(\eta_{nk},\varphi_{nk}\right)+\lambda_{n}-\mu_{nk}}-\frac{I_{mk}+\sigma^{2}}{h_{mnk}}\right]}_{0}^{P_{nk}^{\max}},$$

where $\mu_{nk}=\gamma_{nk}h_{mnk}+\sum_{i=1,i\neq n}^{N}\gamma_{ik}h_{mik}\left[1-2^{\frac{L_{nj}}{B_{0}\left(T_{nj}^{\max}-\frac{C_{nj}}{f_{n}}\right)}}\right]$.

The γ and λ updated by subgradient methods:

(32) $$\gamma_{nk}^{\left(l+1\right)}={\left\{\gamma_{nk}^{\left(l\right)}-\xi_{\gamma }^{\left(l\right)}\times \left[P_{nk}h_{mnk}+\left(1-2^{\frac{L_{nj}}{B_{0}\left(T_{nj}^{\max}-\frac{C_{nj}}{f_{n}}\right)}}\right)\left(I_{mk}+\sigma^{2}\right)\right]\right\}}^{+},$$

(33) $$\lambda_{n}^{\left(l+1\right)}={\left\{(\lambda_{n}^{\left(l\right)}-\xi_{\lambda }^{\left(l\right)}\times \left(P_{nk}^{max}-P_{nk}^{\left(l\right)}\right)\right\}}^{+},$$

where $\xi_{\gamma }^{\left(l\right)}$ and $\xi_{\lambda }^{\left(l\right)}$ represent the step sizes of γ and λ at the $l\in \left\{1,2,\ldots ,L_{\max}\right\}\setminus \left(k-th\setminus \right)$ iteration step, respectively, and $L_{\max}$ is the maximum number of iterations.

The $\xi_{\gamma }^{\left(l\right)}$ and $\xi_{\lambda }^{\left(l\right)}$ should satisfy the following conditions:

(34) $$\sum_{l=1}^{\infty }\xi_{i}^{\left(l\right)}=\infty ,\lim_{l\to \infty }\xi_{i}^{\left(l\right)}=0,i\in \left\{\gamma ,\lambda \right\}.$$

It can be proven that (31) is standard. Therefore, for any value of initial power, the algorithm converges to a unique value. A unique solution can be obtained for (30), so the solution of problem (17) is equivalent to the solution of (30). Algorithm 3 shows the steps of the proposed power allocation algorithm. Correspondingly, the total procedure of the SCORA algorithm is demonstrated in Algorithm 4.
**Algorithm 3** CCCP-based power allocation algorithm.**Input:** The maximum time delay requirement $T_{nj}^{\max}$ for UE *n* to complete the task.**Output:** The optimal power $P^{*}$.1:Initialize l=0 and ε>0. Set $P^{\left(0\right)}$ and calculate $\eta_{nk}^{\left(0\right)}$ and $\varphi_{nk}^{\left(0\right)}$ for all UE devices and MEC servers;2:**while**$\left|\min_{P_{nk}}\frac{1}{N}\sum_{n=1}^{N}\sum_{k=1}^{K}\eta_{nk}^{\left(l\right)}\left[\varphi_{nk}^{\left(l\right)}B_{0}\log_{2}\left(1+\frac{P_{nk}^{\left(l\right)}h_{mnk}}{\sigma^{2}+I_{mk}}\right)-P_{nk}^{\left(l\right)}L_{nj}\right]\right|\geq \varepsilon$ or $l<L_{\max}$ **do**3:    Use CCCP to solve problem (27), and the optimal power $P_{nk}^{*}$ is obtained according to (31);4:    Update $\eta_{nk}^{\left(l+1\right)}$ and $\varphi_{nk}^{\left(l+1\right)}$ according to (24);5:    Update l=l+1;6:**end while**

**Algorithm 4** SCORA algorithm.**Input:** The maximum time delay requirement $T_{nj}^{\max}$ for UE *n* to complete the task.**Output:** Stable matching result $A^{*}$, the subchannel allocation result $Q^{*}$, the computing resources allocation result $F^{*}$ and the optimal power $P^{*}$.
1:Initialize the UE device set N, the MEC servers set M, the subchannel set K and maximum time delay requirement $T_{nj}^{\max}$ for UE device n∈N.2:Obtain the stable UE device and MEC server matching $A^{*}$ according to Algorithm 1.3:Use Algorithm 2 to attain the subchannel allocation result $Q^{*}$ and the computing resources allocation result $F^{*}$.4:Use Algorithm 3 to calculate the optimal power $P^{*}$.


### 4.4. Complexity Analysis

Here, we will analyze the complexity of Algorithm 4, which mainly consists of the following complexities of Algorithms 1–3. In Algorithm 1, the complexity of computing the similarity scores of UE devices is $O\left(n^{2}\right)$. The complexity of each UE device sorting *M* MEC servers in its preference list is $O\left(M\log M\right)$. Therefore, the sorting complexity for *N* UE devices is $O\left(NM\log M\right)$. Considering the worst case, each MEC server will receive *N* requests from UE, and the sorting complexity for *M* MEC servers is $O\left(MN\log N\right)$. Thus, the complexity of Algorithm 1 in the worst case is $O\left(N^{2}+MN\log MN\right)$. In Algorithm 2, for each MEC server, the complexity of comparing task types between $A_{m}$ UE devices and performing the merge operation in the worst case is $O\left({A_{m}}^{2}\right)$. Since the above steps need to be performed for all MEC servers, the complexity of Algorithm 2 is $O\left(M{A_{m}}^{2}\right)$. In Algorithm 3, updating $\left(\eta ,\varphi \right)$ and $\left(\gamma ,\lambda \right)$ requires $L_{\eta \varphi }^{it}$ and $L_{\gamma \lambda }^{it}$ iterations in the worst case, respectively. Hence, the complexity of Algorithm 3 is denoted as $O\left(KMN_{\eta \varphi }^{it}N_{\gamma \lambda }^{it}\log\left(1/\varepsilon \right)\right)$. Therefore, the complexity of the proposed SCORA algorithm can be calculated as $O\left(N^{2}+MN\log MN+M{A_{m}}^{2}+KMN_{\eta \varphi }^{it}N_{\gamma \lambda }^{it}\log\left(1/\varepsilon \right)\right)$.

## 5. Simulation Results

### 5.1. Simulation Setting

In this section, we perform numerical simulations to evaluate the performance of our proposed scheme. The simulation code is written in C language and compiled by Visual Studio 2010. The MEC servers and UE are randomly located in a 100 × 100 m^2^ square area [31], and the simulation parameter settings can be found in Table 2. During subsequent simulations, the ratio of MEC servers to UE is 1:5 if not otherwise specified. To verify the performance of the proposed SCORA algorithm, the following six benchmark schemes are introduced:

- Base scheme: UE devices offload tasks to MEC servers with optimal channel conditions and use maximum transmission power. Subchannels are randomly allocated to UE, and computing resources are equally allocated to UE. Compared with this scheme, the advantages of cooperative task offloading and resource allocation in the proposed SCORA scheme can be demonstrated;
- Non-similarity offloading and power allocation (Non-SOPA) scheme: Compared to the SCORA scheme, this scheme does not consider the task similarity and cooperation-based resource allocation strategy. UE devices offload the tasks to MEC servers with optimal channel conditions, and the transmission power of UE is optimized through the CCCP method. The subchannels and computing resources are respectively allocated to UE devices randomly and equally. In contrast to this scheme, the necessity of considering similarity when offloading tasks and allocating resources can be proven in the SCORA scheme;
- Similarity-based cooperative offloading and CCCP (SCOC) scheme: Compared with the SCORA scheme, this scheme does not consider the cooperation-based resource allocation strategy. Resource allocation is the same as the Non-SOPA scheme. Contrasted against this scheme, the necessity of considering similarity when offloading tasks and allocating resources can be proven in the SCORA scheme;
- Similarity-based cooperative offloading and subchannel allocation (SCOSA) scheme: Compared to the SCORA scheme, this scheme does not consider the CCCP-based power allocation algorithm. UE devices offload tasks to MEC servers with maximum transmission power. In this way, the influence of UE power control on system performance can be reflected;
- Non-energy similarity-based cooperative offloading and resource allocation (Non-ESCORA) scheme: Compared to the SCORA scheme, this scheme does not take into account the UE’s remaining energy levels in either offloading strategy or cluster head selection. Compared to this scheme, the benefits of reducing the energy consumption of UE with low remaining energy levels can be demonstrated;
- Coalitional game-based cooperative offloading (CGCO) scheme [4]: UE devices cooperate to form coalitions to offload their tasks to the MEC servers, and cluster head UE devices are randomly selected. The resource allocation algorithms are the same as in the SCORA scheme. Compared to this scheme, the performance of the cooperative scheme proposed in the SCORA scheme can be evaluated.

**Table 2:** Simulation parameters.

Parameters	Value
The number of MEC servers: *M*	20–50
The number of UE devices: *N*	100–250
The maximum number of UE devices allowed to associate with each MEC server: $A_{m}$	5
The similarity weight: β	0.5
The size of the main code of task *j*: $L_{nj,main}$	[1–10] MB [5]
The size of the task *j* input parameter for UE *n*: $L_{nj,in}$	[0.001–0.01] MB [5]
The number of subchannels: *K*	10
The bandwidth of each subchannel: $B_{0}$	1 MHz
Maximum transmission power of each UE device: $P_{n}^{\max}$	23 dBm [31]
The maximum deadline for task completion: $T_{n}^{\max}$	5 s [20]
Noise power spectral density	−174 dBm/Hz [20]
The CPU cycles required to process each bit of the task	30 cycles/bit
The computing resources of each MEC server	$2.5\times 10^{10}$ cycles/s [5]
Pathloss between MEC server *m* and UE *n*	$128.1+37.6\times \log_{10}\left(d_{mn}\right)$ [36]

### 5.2. Numerical Results

**Figure 4:**
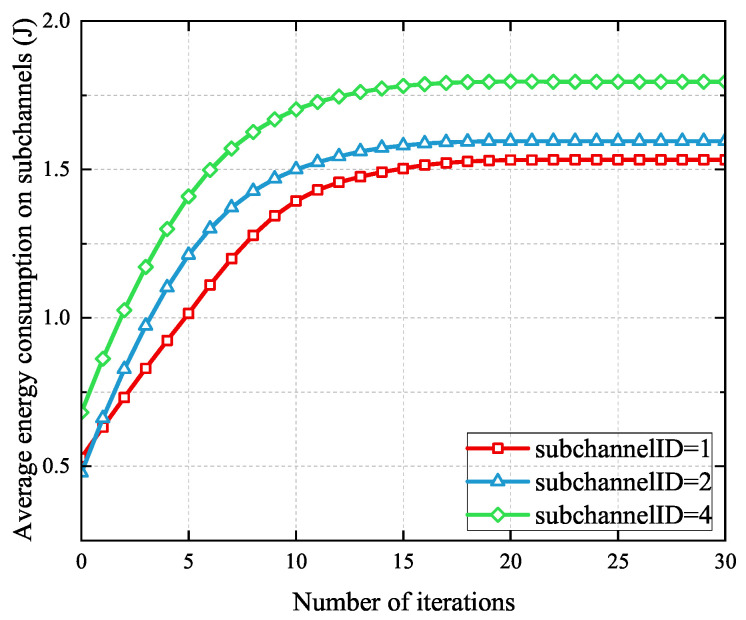
Convergence of the SCORA algorithm.

Figure 4 shows the convergence of the proposed SCORA scheme on different subchannels when there are 20 MEC servers and 100 UE devices. It can be seen that this SCORA algorithm can converge within 20 iterations.

**Figure 5:**
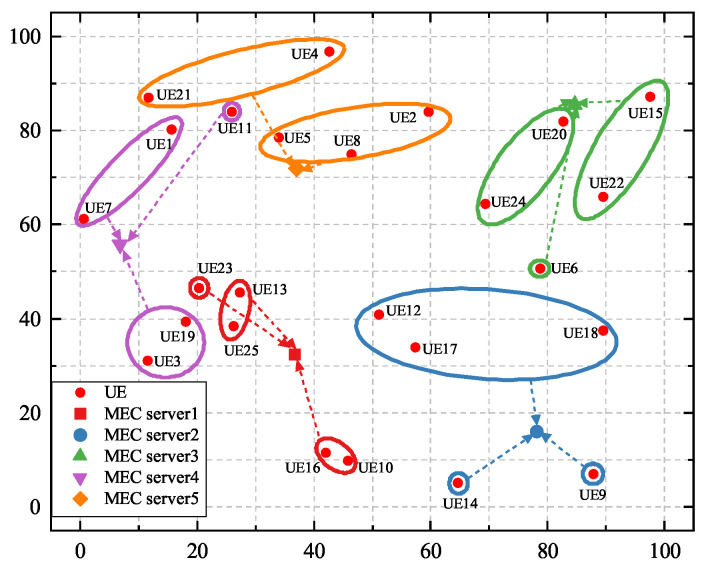
The matching and clustering results between 25 UE devices and 5 MEC servers.

Figure 5 illustrates the similarity-based matching and clustering results when there are 25 UE devices and 5 MEC servers in the system. In Figure 5, UE devices within one circle are assigned to the same cluster, UE devices in circles of different colors have different reusable tasks, and the associations between UE and MEC servers are denoted by dashed arrows. It can be seen that a large number of UE devices are connected to their nearest MEC servers. However, there are a few UE devices that are not matched to the nearest MEC servers. For example, instead of connecting to the nearest option, MEC server 1, UE 12 connects to MEC server 2. This is because UE 12 has the same task type as UE 17 and UE 18. Therefore, they form one cluster in order to conduct cooperative offloading.

**Figure 6:**
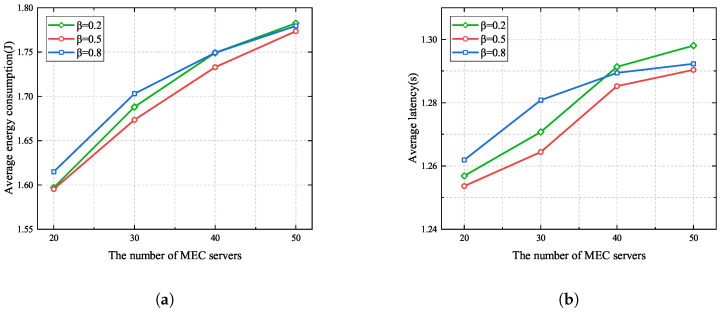
Different similarity score weights in the proposed algorithm. (**a**) Average energy consumption. (**b**) Average latency.

Figure 6 presents the impacts of different similarity score weights β when the number of MEC servers ranges from 20 to 50. It can be seen that average energy consumption and latency both increase with the number of MEC servers. This is because the number of UE devices scales linearly with the number of MEC servers, resulting in more energy consumption and more severe interference, which reduces the task uploading rate. In addition, it can be observed that when the number of MEC servers is less than 40, β=0.2 yields better performance than β=0.8. This is due to the fact that UE devices offload their tasks to the MEC servers with better channel conditions and obtain a high task offloading rate when β=0.2. However, when the number of MEC servers is more than 40, fewer UE devices tend to cooperate, and more UE devices need to offload their main codes to the MEC servers when β=0.2, leading to greater latency and more energy consumption when β=0.8. Moreover, from Figure 6, we can see that the average energy consumption and latency when β=0.5 are less than those when β=0.2 and β=0.8. This is because the similarity score and channel gain are balanced when β=0.5; thus, UE devices with good channel conditions can cooperate to offload their tasks.

**Figure 7:**
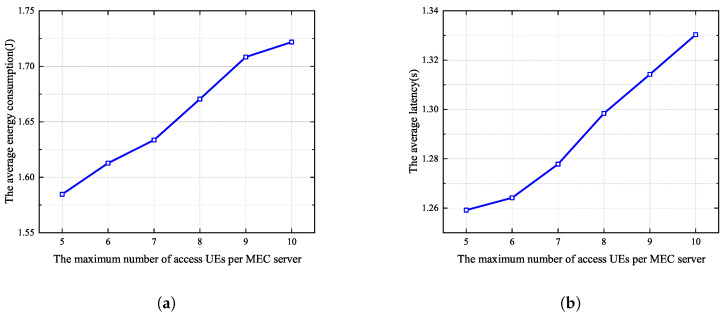
Different maximum numbers of UE devices allowed to associate with each MEC server in the proposed algorithm. (**a**) Average energy consumption. (**b**) Average latency.

Figure 7a and Figure 7b respectively show the average energy consumption and delay when the maximum number of UE devices allowed to associate with each MEC server changes from 5 to 10. In this simulation, the total number of UE devices in the MEC system is 100, and the number of MEC servers is 20. It can be seen that as the maximum number of UE devices grows, both energy consumption and latency gradually increase. The reason for this is that more cooperative UE devices offload their tasks to the same MEC server, leading to an increase in the number of cooperating clusters and increased competition for spectrum resource, resulting in an increase in the average energy consumption and delay. Therefore, in the subsequent simulations, we set the maximum number of UE devices that can access each MEC server $A_{m}$ to 5.

**Figure 8:**
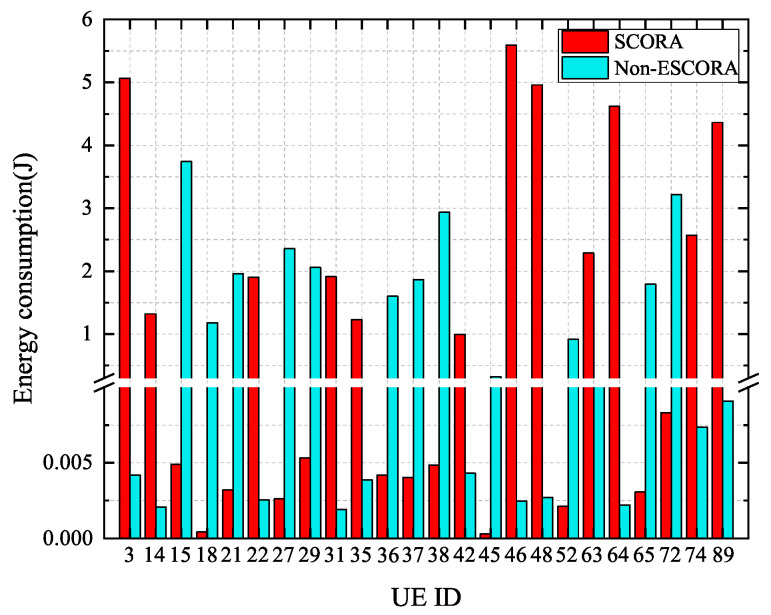
Comparison of the energy consumption of cluster head UE devices in different schemes.

Figure 8 shows the energy consumption of cluster head UE devices in the SCORA and Non-ESCORA schemes. The remaining energy levels of cluster head UE devices are presented in Table 3. For the sake of clarity, only different cluster head UE devices’ information from the two schemes is given in Table 3, ignoring information the same cluster head UE devices. From Figure 8 and Table 3, it can be seen that the cluster head UE devices consume more energy compared to non-cluster head UE in all schemes. The cluster head UE devices chosen by the SCORA scheme have more remaining energy than those selected by the Non-ESCORA scheme. The reason for this is that the remaining energy factor is considered in the SCORA scheme, which helps to reduce the energy consumption of UE with low remaining energy levels.

**Figure 9:**
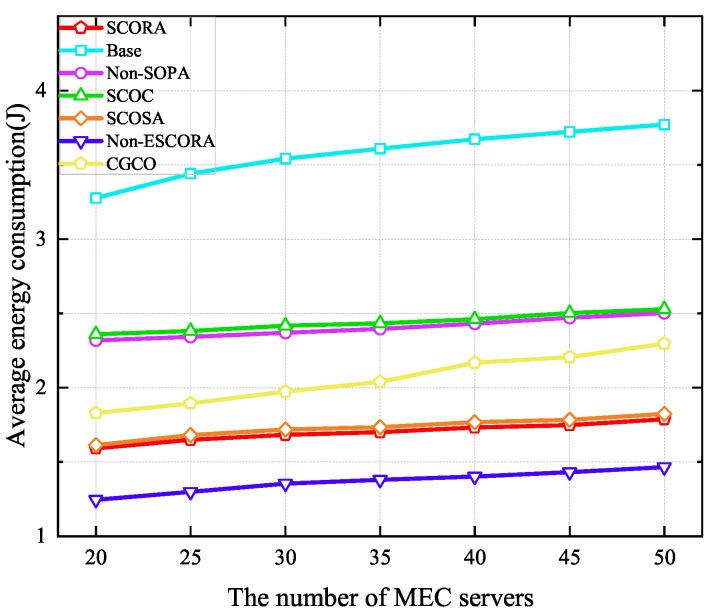
Average energy consumption versus the number of MEC servers.

Figure 9 depicts the average energy consumption of UE under a varying number of MEC servers. It can be seen that average energy consumption increases with the number of MEC servers due to the increase in the number of UE devices. Compared to the Base and SCOSA schemes, the proposed SCORA algorithm can reduce the average energy consumption by 51.52% and 1.24%, respectively, when the number of MEC servers is 20. This proves that the proposed offloading strategy, resource allocation strategy, and CCCP-based power allocation can be efficient in saving UE energy. However, since the remaining energy levels of the UE are taken into account in both the matching strategy and the selection of cluster head UE, in the proposed scheme, many UE devices matched with MEC servers without the best channel conditions, as the trade-off between the UE’s remaining energy level and channel conditions is considered when choosing the cluster head UE. Therefore, the SCORA scheme consumes more energy than the Non-ESCORA scheme. Although the CGCO scheme adopts cooperative task offloading, it exhibits higher average energy consumption than the SCORA scheme due to its random cluster head selection strategy.

**Figure 10:**
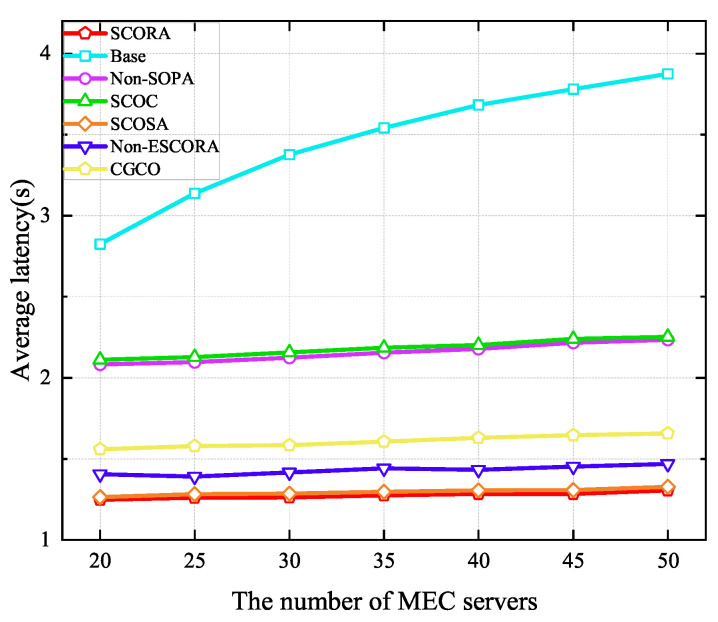
Average latency versus the number of MEC servers.

Figure 10 shows the average latency of UE when the number of MEC servers ranges from 20 to 50. It can be seen that the average latency of UE in all schemes increases with the growth of the MEC server density due to the severe interference caused by the increase in the number of UE devices. Moreover, we can see that our proposed scheme is able to obtain lower delay than others. Specifically, compared to the Base and SCOSA schemes, the latency of UE in the SCORA scheme is reduced by 0.79% and 55.83%, respectively, when the number of MEC servers is 20. This is due to the fact that more subchannels and computing resources are assigned to the cluster head UE devices, resulting in a higher task transmission rate. From Figure 9 and Figure 10, we can see that the SCORA scheme obtains good performance in terms of UE energy savings and task latency reduction. Therefore, our proposed SCORA scheme can improve the efficiency of the entire system.

**Figure 11:**
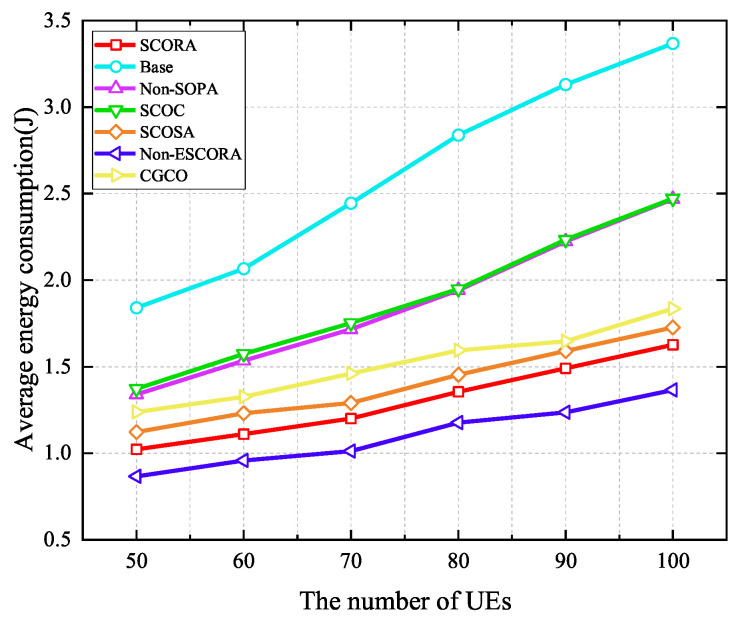
Average energy consumption versus the number of UE devices.

**Figure 12:**
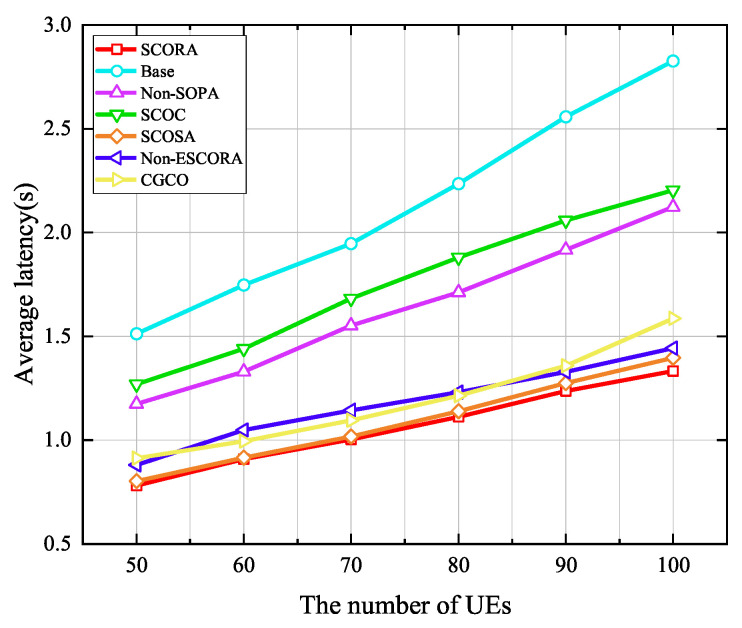
Average latency versus the number of UE devices.

Figure 11 depicts the average energy consumption of UE given a varying number of UE devices when the number of MEC servers is 20. It can be seen that average energy consumption increases with the growth of the number of UE devices due to the fact that more tasks need to be offloaded. In addition, we can observe that the proposed SCORA scheme performs better in reducing the UE’s energy consumption compared to other schemes such as Base, Non-SOPA, SCOC, SCOSA, and CGCO. This indicates that the SCORA scheme effectively improves the resource utilization efficiency and reduces the overall energy consumption of the UE by jointly optimizing the task offloading, resource allocation, and power allocation. However, the SCORA scheme results in higher energy consumption than Non-ESCORA. The reason for this is that UE devices with more remaining energy are selected as the cluster head UE to transmit the main codes to the MEC servers in the SCORA scheme, while the UE devices with the best channel conditions are chosen as the cluster head UE in the Non-ESCORA scheme. Therefore, the UE in the SCORA scheme should upload the main codes with higher power in order to meet the UE devices’ latency requirements. In Figure 12, although the average latency in the SCORA scheme is reduced compared to the Non-ESCORA scheme, the energy consumption is increased due to the use of higher transmission power.

The effect of the UE density on the average latency is evaluated in Figure 12, where the number of MEC servers is 20. It can be observed that the average latency of UE in all schemes increases with the growth of the UE density due to the severe interference caused by the increase in the number of UE devices. Compared to all the other schemes, the SCORA scheme attains the lowest average delay, which is mainly due to the fact that the SCORA scheme allocates more subchannels and computing resources to UE devices with the same tasks through a cooperative offloading strategy, thus improving the speed of task transmission and processing. From Figure 11 and Figure 12, we can see that as the number of UE devices increases, compared with other schemes, the SCORA scheme can significantly reduce the average energy consumption and latency of UE. Therefore, our proposed SCORA scheme can work well in a relatively DECS.

Table 4 shows the energy consumption and latency data for each scheme and the energy consumption and latency gains obtained by the proposed SCORA compared to the six benchmark schemes when the number of MEC servers is 20, and the number of UE devices is 100. It can be seen that the proposed SCORA algorithm has significant advantages for energy consumption and latency reduction.

## 6. Conclusions

In this study, we focus on addressing the energy consumption challenge in DECS with reusable tasks while considering the task similarity between UE devices. By jointly optimizing task offloading, resource allocation, and power allocation, the problem is formulated as an MINLP problem, and the SCORA scheme is proposed to solve it. This strategy aims to minimize the total energy consumption of all UE while considering the remaining energy levels of UE. In the SCORA scheme, the similarity-based matching offloading strategy is developed to solve the task offloading subproblem, the cooperation-based resource allocation strategy is proposed to solve the subchannel and computing resource allocation subproblem, and the CCCP-based power allocation strategy is proposed to solve the UE transmission power optimization subproblem. Through extensive simulation experiments, the SCORA scheme was compared to six benchmark approaches. The results demonstrate that the SCORA scheme can significantly outperform existing schemes in terms of reducing the energy consumption of UE. Notably, compared to the scheme neglecting UE’s remaining energy levels, the SCORA scheme exhibits remarkable advantages in terms of energy conservation for UE with low remaining energy levels, highlighting its adaptability to UE heterogeneity. These findings indicate that the proposed SCORA scheme can provide an efficient and practical solution for joint task offloading and resource allocation in DECS. Since the task offloading in a dynamic environment is more complicated, we consider the computation offloading in a static environment and assume that the user’s task is indivisible. In the future, with the help of the deep reinforcement learning method, our work will expand to intelligent task offloading and resource allocation problems in a dynamic MEC network environment where UE mobility will be taken into account.

## 7. Patents

An invention patent entitled “A Task Similarity-Based Task Offloading Method, System, and Storage Medium” based on the content of this manuscript has been submitted to the China National Intellectual Property Administration. The invention patent application number is 202510264933.X.

## Figures and Tables

**Table 1:** List of main symbols.

Symbol	Definition
M	The set of MEC servers
N	The set of UE devices
K	The set of subchannels
$L_{nj}$	The total size of task *j* that is offloaded by UE *n*
$L_{nj,main}$	The main code size of task *j* that is offloaded by UE *n*
$L_{nj,in}$	The input parameters size of task *j* that is offloaded by UE *n*
$C_{nj}$	The CPU cycles required to complete the task *j* that is offloaded by UE *n*
$C_{nj,main}$	The CPU cycles required to complete the main code of task *j* that is offloaded by UE *n*
$C_{nj,in}$	The CPU cycles required to complete the input parameters of task *j* that is offloaded by UE *n*
$T_{nj}^{\max}$	The latency requirement for UE *n* to complete the task *j*
$B_{0}$	The bandwidth of each subchannel
$R_{n}$	The uplink transmission rate of UE *n*
$T_{nj}^{t}$	The transmission latency of UE *n* for offloading
$T_{nj}^{s}$	The computational latency of task *j* that is offloaded by the UE *n*
$E_{nj}^{t}$	The transmission energy consumption of UE *n* for offloading task *j*
$f_{n}$	The computing resource allocated to UE *n*
$P_{n}^{\max}$	The maximum transmission power of UE *n*

**Table 3:** The remaining energy of cluster head UE devices in the same cluster for different schemes.

SCORA Cluster Head UE ID	3	14	22	31	35	42	46	48	63	64	74	89
remaining energy levels	0.90	0.98	1.00	1.00	0.67	0.82	1.00	0.89	1.00	0.82	0.46	0.78
Non-ESCORA cluster head UE ID	72	45	29	21	52	27	38	15	65	36	18	37
remaining energy levels	0.78	0.14	0.94	0.90	0.18	0.42	0.52	0.67	0.32	0.67	0.21	0.33

**Table 4:** Performance of the different schemes and the gains obtained by the proposed SCORA scheme.

×	SCORA	Base	Non-SOPA	SCOC	SCOSA	Non-ESCORA	CGCO
Energy consumption (J)	1.59	3.28	2.32	2.36	1.61	1.25	1.83
Latency (s)	1.25	2.83	2.08	2.11	1.26	1.40	1.56
Energy consumption gains	×	51.52%	31.47%	32.63%	1.24%	–27.20%	13.11%
Latency gains	×	55.83%	39.90%	40.76%	0.79%	10.71%	19.87%

## Data Availability

Data are contained within the article.

## Abbreviations

The following abbreviations are used in this manuscript:

MEC	Mobile edge computing
CCCP	Convex–concave procedure
IoT	Internet of things
UE	User equipment
QoE	Quality of experience
ETSI	European Telecommunications Standards Institute
MCC	Mobile cloud computing
CPU	Central processing unit
DECS	Dense edge computing systems
UDN	Ultra-dense network
CSBL	Contextual sleeping bandit learning
CSBL-M	CSBL-multiple
PSO	Particle swarm optimization
EH	Energy harvesting
SBSs	Small base stations
NOMA	Non-orthogonal multiple access
WPT	Wireless power transfer
UAV	Unmanned aerial vehicle
OFDMA	Orthogonal frequency division multiple access
MINLP	Mixed-integer nonlinear programming

SCORA	Similarity-based cooperative offloading and resource allocation
Non-SOPA	Non-similarity offloading and power allocation
SCOC	Similarity-based cooperative offloading and CCCP
SCOSA	Similarity-based cooperative offloading and Subchannel Allocation
Non-ESCORA	Non-energy similarity-based cooperative offloading and resource allocation
CGCO	Coalitional game-based cooperative offloading
